# Interaction between Vestibular Compensation Mechanisms and Vestibular Rehabilitation Therapy: 10 Recommendations for Optimal Functional Recovery

**DOI:** 10.3389/fneur.2014.00285

**Published:** 2015-01-06

**Authors:** Michel Lacour, Laurence Bernard-Demanze

**Affiliations:** ^1^Laboratoire de Neurobiologie Intégrative et Adaptative, UMR 7260 CNRS/Université Aix-Marseille, Fédération de Recherche 3C, Centre de St Charles, Marseille, France; ^2^Service d’otorhinolaryngologie et d’otoneurologie, CHU Nord, Assistance Publique-Hôpitaux de Marseille, Marseille, France

**Keywords:** vestibular compensation mechanisms, vestibular rehabilitation therapy, critical period, adaptation, habituation, anxiety and stress, motivation, ecologic contexts

## Abstract

This review questions the relationships between the plastic events responsible for the recovery of vestibular function after a unilateral vestibular loss (vestibular compensation), which has been well described in animal models in the last decades, and the vestibular rehabilitation (VR) therapy elaborated on a more empirical basis for vestibular loss patients. The main objective is not to propose a catalog of results but to provide clinicians with an understandable view on when and how to perform VR therapy, and why VR may benefit from basic knowledge and may influence the recovery process. With this perspective, 10 major recommendations are proposed as ways to identify an optimal functional recovery. Among them are the crucial role of active and early VR therapy, coincidental with a post-lesion sensitive period for neuronal network remodeling, the instructive role that VR therapy may play in this functional reorganization, the need for progression in the VR therapy protocol, which is based mainly on adaptation processes, the necessity to take into account the sensorimotor, cognitive, and emotional profile of the patient to propose individual or “à la carte” VR therapies, and the importance of motivational and ecologic contexts. More than 10 general principles are very likely, but these principles seem crucial for the fast recovery of vestibular loss patients to ensure good quality of life.

## Background

The vestibular system was originally thought to contribute to reflex generation for posture (1) and oculomotor (2) control, in close convergence with other sensory and motor signals (3). More recently, the vestibular system has been recognized for its contribution to, and interaction with, high-level cognitive processes including space perception (4), spatial navigation (5), body representation (6), attention (7), memory (8), mental imagery (9), and even social cognition (10).

The dramatic consequences of vestibular disorders therefore incorporate a wide range of symptoms including loss of balance, blurred vision, and vertigo, as a result of impaired vestibulo-spinal and vestibulo-ocular reflexes (VORs) and of abnormally activated vestibulo-thalamo-cortical pathways, respectively (11, 12). Impaired inputs to the vestibular cortex and hippocampus very likely explain the deficits in spatial navigation and memory tasks (13, 14) as well as in body representation and bodily self-consciousness (10). The functional, psychological, and social impacts of vestibular impairment are very important from a societal point of view. A vertigo crisis, together with the physical disability and the psychological stress that accompany a vestibular disorder, results in socio-professional consequences (stop working), psychological and social isolation (15) in many cases. As a rule, the vestibular syndrome is ameliorated over weeks and months in both humans and animals through the process of vestibular compensation [(16–18), for reviews].

The recovery of the static symptoms, observed in a stationary patient, must be differentiated from the recovery of the dynamic symptoms, seen only when the patient moves his/her head or his/her whole body in space (19). The static symptoms incorporate spontaneous vestibular nystagmus, skew deviation and eye cyclotorsion (oculomotor signs), head and body tilt (vestibulo-spinal signs), vertigo, and modification of the subjective visual vertical (SVV) (perceptual and orientation signs), usually aggravated by neurovegetative disorders (nausea and vomiting). This static syndrome is generally fully compensated for with a relatively short time constant depending on the species: a few days (rat model), weeks (cat model), or months (humans). In contrast, the dynamic deficits (drops in gains and phase shifts of the VOR, reduction of the time constant of the VOR on the affected side, and impaired balance control in challenging contexts) remain poorly compensated and are exhibited over a longer time period. Moreover, in many cases, the VOR does not recover at all. The permanent deficit of dynamic VOR function was first described by the Australian group in vestibular loss patients during passive head impulses using short-duration angular accelerations in the natural range of head motion (2000°–3000°/s^−2^), directed toward the affected side [Head Impulse Test: HIT, see Halmagyi et al. (20)]. In such situations, a compensatory catch-up saccade can be observed after the end of head rotation, indicating that the canal response is lacking. However, a permanent deficit can be masked by sensory or behavioral strategies. A good illustration is the covert saccade described by the same Australian group during brief, passive, unpredictable head impulses to the lesion side (21, 22).

Vestibular compensation therefore includes a rapid vestibulo-centric static process and a longer term, dynamic, distributed learning process (19). We have recently proposed the dual concept of brain orchestration of “neurobiological melodies” and “behavioral melodies” to explain the recovery of the static and dynamic functions, respectively (23). The former strongly depends on the vestibular etiology, while the latter depends on the patients themselves. Depending on the nature of the vestibular damage (sudden and total versus progressive and partial, reversible or not), the neurobiological orchestration occurring in the deafferented vestibular nuclei (VN) was drastically different (24–26). For example, a cell proliferation and cell differentiation process was found in the deafferented VN only after an acute and total deafferentation, and never after a peripheral labyrinthectomy or a reversible blockade of the action potentials in the vestibular nerve by intra-tympanic injection of tetrodotoxin. That means that the plastic events in the deafferented VN strongly differ depending on the vestibular pathology [vestibular neuritis, Menière’s disease, vestibulotoxic antibiotics, benign paroxysmal positional vertigo (BPPV), and the aging process]. On the other hand, the sensory and behavioral strategies substituting for the missing dynamic vestibular functions show great inter-individual variability. To avoid retinal slip and oscillopsia during head rotation to the diseased side, some patients close their eyes or use blinks, others keep the head facing the same direction as the trunk and turn slowly, while others pre-program a saccade. Such vicariant idiosyncratic strategies, depending on the patients themselves, are of clinical relevance for the physiotherapist who must help the patient to use the best strategy and to quickly obtain a good quality of life.

## Why and How Can Vestibular Compensation be Improved?

There are several reasons to search for methods to improve the consequences of vestibular damage. One is the high prevalence of vestibular dysfunctions, which alters balance control and can lead to falls, in the general population. Data from the 2001–2004 National Health and Nutrition Examination Survey collected in 5086 US adults aged 40 years and older indicate that 35.4% had vestibular dysfunction (27). This percentage increased significantly with age, reaching 64.8% in people over 60 and 84.8% in people over 80. The increased risk of fall with age is among the most morbid and costly health conditions affecting older individuals in modern societies.

A second reason to manage vertigo and dizziness is because of their strong impact on the quality of life. In a telephone survey of 1003 patients with moderate to severe vertigo or dizziness, 80% reported the need for medical consultation, and vertigo and dizziness interrupted their daily activities (28). Vertigo involves not only physical factors, but also emotional factors that are extremely disabling (nausea, oscillopsia), and increase the tendency of falling. In addition, anxiety and depression are increased in patients with vestibular dysfunction and vertigo (29, 30).

Another reason for enhancing functional vestibular compensation is that the spontaneous or “naturalistic” recovery is not optimal. For example, patients can replace some lost vestibular functions (the VOR) by new maladaptive strategies (limitations of head movements). As stated in their review on the translational lessons from vestibular rehabilitation (VR) (31), the main goal for VR therapy is to improve the dynamic performances by new, learned strategies that lead to the best optimal functional recovery. Finding an optimal rehabilitation paradigm will not only accelerate the time-course of recovery but will also achieve the best selection of strategies for regaining a better quality of life.

One can distinguish two broad paths in the management of vertigo and dizziness patients. One is drug-based medicine with the discovery of pharmaceutical agents acting on different targets known to influence the recovery process after vestibular injury. Neuropharmacology of vertigo and dizziness, recently reviewed by Soto and Vega (32), will not be covered in this review. The other is behavioral-cognitive therapy, which has been empirically elaborated and developed in many ways for addressing the different facets of vestibular syndrome. This second path originates with the early twentieth century discovery of clinicians and behavioral scientists, who showed empirically or by more experimental investigations, respectively, that behavioral therapies could improve recovery from vestibular damage. The term “VR” was assessed for the vestibular loss patients, as compared with the “rehabilitative training” used for stroke patients. According to the international classification of functioning disability [Geneva: (33)], rehabilitation as a goal “to reach and maintain optimal functioning in physical, intellectual, psychological, and/or social domains” is evidence based medicine.

The concept of “VR” was formalized at the end of the second world war by two British practitioners, Sir Terence Cawthorne and F. S. Cooksey, who developed a balance rehabilitation strategy for injured British soldiers (34, 35). This concept was highlighted and updated in more recent publications and reviews by different groups throughout the world, which have proposed different VR therapy protocols, strategies, and exercises for improving the functional recovery of the vestibular syndrome, and more generally for rehabilitating dizzy patients (18, 31, 36–38). The Cochran Database of Systematic Reviews in Ref. (39) indicates that “there is moderate to strong evidence that VR is a safe, effective management for unilateral peripheral vestibular dysfunction, based on a number of high quality randomized controlled trials.” The more recent Cochran Database of Systematic Reviews published in 2011 has confirmed these conclusions (40).

The main goal of the review is to question how the brain-plasticity mechanisms responsible for the spontaneous recovery can help VR therapy, and how VR therapy can interact with these basic mechanisms. Thus, there is a need for clarification to assist the professional community, to change traditional clinical strategies used by some professional therapy practitioners, and to help them in finding an optimal rehabilitation paradigm. Among the most relevant questions for practitioners are the following: When is early enough? Are there windows of opportunity? What type of VR? How long? Can VR alter the functional neuronal reorganization? Why do some patients have a poor recovery, or decompensate? …

## Ten Recommendations for Optimal Functional Recovery

The purpose here is neither to provide a rigid guideline for VR therapy nor to compare the efficacy of different rehabilitation techniques, or even to propose the optimal VR paradigm, but to provide the whole community of practitioners and physiotherapists with understandable principles to guide their VR therapy. We have regrouped these principles required for optimal functional recovery after vestibular injury under the term “ten recommendations” (just a wink to Moses), but more than 10 are likely to exist.

### Engage vestibular rehabilitation behaviorally (favor active retraining)

The explosively developing scientific domain of the integrative and cognitive neurosciences seen during the last decades revealed first that the brain is continuously plastic [(41, 42) for reviews]. Brain remodeling can be induced at any age, and the neuronal reorganizations are experience-dependent. The mechanisms of experience-dependent plasticity clearly contribute to post-stroke brain reorganization and to the efficacy of rehabilitative training [(43) for review]. The plastic remodeling of the synaptic connections obeys the Hebbian principle of neuronal network plasticity, which tells us that when the brain is engaged behaviorally the inputs activated simultaneously in time strengthen together and increase their cooperativity (44). This Hebbian law must apply to brain injury in general (45), and to the VR therapy-induced plasticity changes in particular. Functional recovery should depend on the therapeutic training regime, which is expected to be better in active compared with passive vestibular loss patients. Active training and physical activity have been shown to induce structural and functional neuronal reorganizations together with behavioral and cognitive improvements in animal models, compared with untrained individuals (46). In other words, a patient who is engaged behaviorally, and actively, will recover faster than a patient who watches TV all day in a chair.

**Figure 1:**
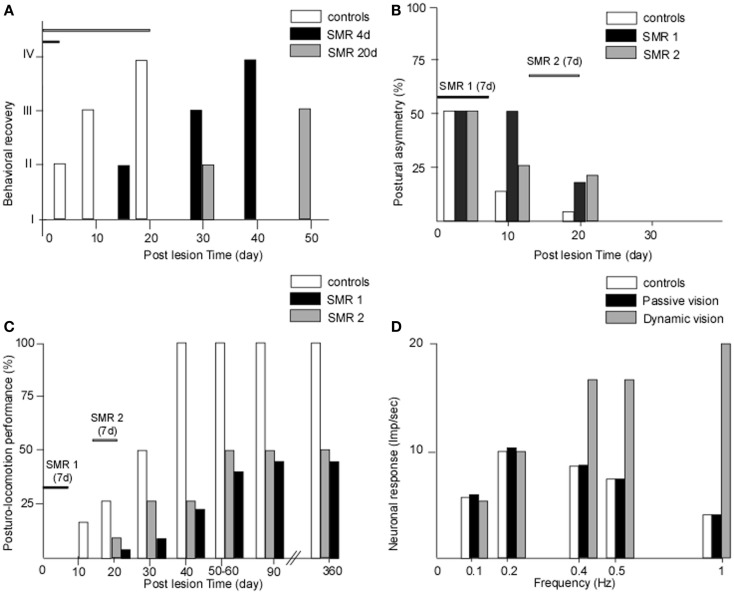
**(A–D) The crucial role of early and active retraining in animal models of vestibular loss**. Data from animal models showing the effects of restraining the post-lesion sensorimotor activity. **(A)** The effects of sensorimotor restriction (SMR) applied just after a unilateral vestibular neurectomy (UVN) in the monkey (baboon: Papio papio) for a short (4 days) or longer (20 days) time duration on the behavioral state of recovery (states I–IV for maximal to minimal posture-locomotor deficits, respectively: ordinates). The behavioral recovery was frozen as long as the SMR was applied, and the time to full recovery was strongly delayed compared with unrestrained animals, the more so the longer the SMR [modified from Lacour et al. (47)]. **(B,C)** The effects of 1 week of SMR applied at different time windows after UVN on the recovery of posture **(B)** and of posturo-locomotor performance **(C)** in the cat model. The SMR was applied very early after UVN (SMR 1: day 3 to day 9) or later during the compensatory stage (SMR 2: day 14 to day 20). Postural asymmetry **(B)** as well as dynamic equilibrium **(C)** were strongly delayed in the cats submitted to SMR1 and SMR2 compared with unrestrained animals. More drastic effects were observed for the dynamic equilibrium function with a final level of recovery 1 year post-lesion limited to 40 and 50% of the preoperative maximal performance for the SMR 1 and SMR 2, respectively [modified from Xerri and Lacour (48)]. **(D)** The role of dynamic visual cues on the neuronal response of vestibular nuclei cells to optokinetic stimulation in the UVN cat. In the intact animal, the neuronal response (in impulses per second) was limited to the low frequency range of optokinetic stimuli (0–0.5 Hz). In the UVN cats submitted to passive visual optokinetic stimulation, there was no change compared with the controls, while the UVN cats dynamically receiving the optokinetic stimulation showed strongly increased neuronal responses, the more so the higher the frequency. In cats moving freely in their optokinetic environment, the visual cues substituted for vestibular input at high frequencies, up to 1 Hz, a result never seen in intact cats, which indicates that the post-lesion experience may determine new neuronal properties that alter the recovery process [modified from Zennou-Azogui et al. (49)].

We provided the first experimental demonstration in monkeys that the recovery process can be totally blocked when submitting baboons to sensorimotor restriction just after vestibular injury (47). In this pioneering work, we developed the sensorimotor restriction paradigm with the goal of reproducing the behavior of Menière’s disease patients who are candidates for a curative vestibular neurectomy, who observed in France for 2 weeks under bed-rest after surgery that induced a lack of behavioral sensorimotor activity. Figure 1A shows the behavioral recovery in three groups of baboons receiving unilateral vestibular neurectomies. Group 1 was free to move in their natural environment while the two other groups were submitted to sensorimotor restriction applied just after surgery, which consisted in immobilizing the animals in a primate chair for 4 days (group 2) or 20 days (group 3). The results showed that group 1 recovered very fast, within 3 weeks, and that the sensorimotor restriction had totally frozen the recovery process in the two other groups. In groups 2 and 3, the animals exhibited the typical vestibular syndrome (as seen just after surgery) when they were replaced in their natural environment, without exhibiting any sign of recovery. Additionally, the time-course of their behavioral recovery was strongly delayed later on, and was greater the longer the sensorimotor restriction was enforced. Similar results were found in a cat model, using the same experimental protocol with 1 week of sensorimotor restriction, which consisted of keeping the cat in a small box and preventing the animal from standing erect and moving [(48), cf. Figure 1B].

These data, which clearly indicated that active behavioral engagement is necessary to start the vestibular compensation process, have constituted the starting point of VR in France and other European countries. Active rehabilitation feeds the central nervous system with all the normal sensorimotor cues and regulates vestibular function in healthy subjects (posture orientation and stabilization, gaze stabilization, verticality perception, and spatial navigation). In vestibular loss patients, these inputs participate in feedback and feed forward mechanisms used to recalibrate the impaired vestibular functions. Recent investigations have attempted to identify the molecular and cellular changes in the central nervous system in response to physical activity. Several key responses have been identified in animal models, including the up-regulation of neurotrophins, increased neurogenesis, and improved learning and memory. Exercise therefore constitutes a behavioral intervention to promote brain health, plasticity, and functional recovery (46).

Taken together, these data corroborate the general statement that adaptation processes, which require dynamic interactions between the subject and the environment, are the main basis of learning and restoration of brain functions. One must therefore question the therapeutic protocols based only on passive exercises and habituation (see Favour Adaptation Rather than Habituation Processes).

### Begin vestibular rehabilitation early (the concept of the sensitive period)

The role of experience in the developing brain has been understood for a long time, and the literature shows higher experience-dependent plasticity during early sensitive periods. External stimuli shape the neural circuitry patterns using synaptic competition mechanisms, with the most activated neural connections being validated and those less activated being eliminated. During the early period of brain development, manipulations of the environmental context considerably alter the neural connectivity and the functional properties of neurons, as demonstrated in the visual cortex by the Nobel Prize winners Hubel and Wiesel (50). The major connectivity patterns can be refined continuously across the lifespan, through experience and learning. One important question for VR therapy is to know if such sensitive periods exist after vestibular injury and constitute windows of opportunity for physiotherapists. In the stroke domain, several studies showed that rehabilitative training was more effective if initiated early after stroke. In the monkey model, beginning training within 1 week after motor cortical infarct spared the paretic hand representation in the motor cortex, an effect that was lost if training was delayed until 30 days post-infarct (51). Thus, is there a critical plastic window in the vestibular compensation process?

In our first proof of concept studies of the critical or sensitive period, we have applied the sensorimotor restriction paradigm to unilateral vestibular neurectomy (UVN) cats and investigated the effects of a 1-week restriction, distributed at different time periods after vestibular lesion, on the static and dynamic control of posture and gait (48). A 1-week sensorimotor restriction applied during the acute stage (first post-lesion week) or during the early stage of compensation (third post-lesion week) had dramatic effects on the time-course of recovery. Not only were the static and dynamic equilibrium performances of the cats strongly delayed compared with the UVN cats without restriction, but the final level of recovery was drastically reduced (40 and 50% of the performance of unrestrained animals, respectively: cf. Figures 1B–C). When applied in a totally compensated cat, at 6 weeks post-lesion, the sensorimotor restriction had no effect. A comparison of Figures 1B,C shows that the more drastic effects of the sensorimotor restriction are on the dynamic equilibrium function recovery.

**Figure 2:**
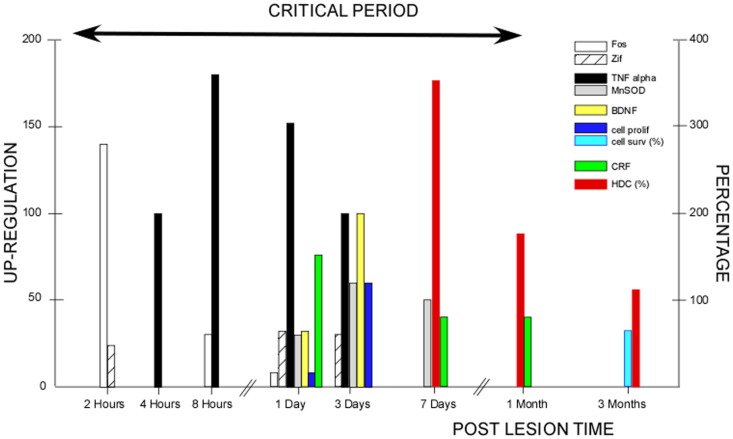
**Post-lesion vestibular plasticity and vestibular rehabilitation therapy: inter-relations during the critical period**. This figure illustrates the plastic events occurring in the vestibular nuclei (VN) after unilateral vestibular deafferentation in animal models. The first event was the up-regulation of immediate early genes (IEGs) in the very early hours and days, with Fos immunoreactivity peaking 2 h post-lesion (open histograms) and Zif-268 immunoreactive neurons peaking 1–3 days post-lesion (hatched histograms). Both IEG expressions declined progressively within 3 days to 1 week [modified from Gustave Dit Duflo et al. (53) and Lacour and Tighilet (24)]. Many plastic events showed up-regulation peaking at 3 days. Neurotrophin immunoreactivity occurred 1 day post-lesion, with peak expression of the brain-derived nerve factor (BDNF: yellow histograms) at 3 days in the VN and related structures, and a return toward basal expression within the first post-lesion week. Similar spatio-temporal patterns were found for the nerve growth factor, the neurotropin 3, and their respective TrKA/TrKC receptors [modified from Lacour and Tighilet (24)]. Using bromodeoxyuridine (BrdU) as a newborn cell marker, intense cell proliferation was found in the deafferented VN after total and sudden unilateral loss of vestibular function, with a peak of cell proliferation at 3 days (dark blue histograms). Later on, cell proliferation was followed by cell differentiation (GABAergic neurons, microglial cells, and astrocytes). At 3 months, 70% of the newborn cells survived (light blue histograms) [modified from Tighilet et al. (54) and Lacour and Tighilet (24)]. Immunolabeling of markers of inflammatory responses such as tumor necrosis factor alpha (TNF alpha: black histograms), and of markers of neuroprotection such as manganese superoxide dismutase (MnSOD: gray histograms), showed up-regulations detectable as early as 4 h post-lesion, peaking at 8 h to 1 day and regaining normal values at 3 days to 1 week for TNF alpha. A more delayed expression for MnSOD was observed at 1 day, peaking at 3 days and returning to control values at 15 days [modified from Liberge et al. (55)]. Plasticity of the hypothalamo-pituitary-adrenal axis (HPA or stress axis) was characterized by an increased immunostaining for corticotrophin-releasing factor (CRF) and arginine vasopressine in the paraventricular nucleus of the hypothalamus. CRF up-regulation was observed as early as 1 day and persisted during the whole compensatory stage, until 1 month post-lesion (green histograms). An opposite pattern with down-regulation of the number of CRF-immunoreactive neurons was seen in the VN (not illustrated). The long-lasting activation of the HPA axis reflects chronic stress that was no longer present when the animals were totally compensated at 3 months [modified from Tighilet et al. (56)]. Increased histidine decarboxylase (HDC) mRNA expression in the TM nuclei was observed acutely with a peak at 7 days (350% of the basal level) and a return toward control values at 3 months (red histograms). HDC up-regulation during the whole compensatory stage (the enzyme synthesizing histamine) points to the role of the histaminergic system in behavioral recovery [modified from Tighilet et al. (57) and Lacour and Tighilet (24)]. The up-regulation of the GABAergic and cholinergic systems (not illustrated) found in the acute (at 1 week) and chronic (at 3 months) stages of vestibular compensation supports the idea that they play a significant role in the maintenance of compensation in totally compensated animals. All of the plastic events occurring during the first post-lesion month are indicative of a critical period (arrow), which should benefit vestibular rehabilitation and during which vestibular plasticity could be shaped by vestibular rehabilitation therapies.

The critical period we have demonstrated in our cat model covers the whole first post-lesion month. This time window coincides with the various plastic reorganizations occurring in the VN and associated neuronal networks (24) (Figure 2). Most of the basic mechanisms expressed in the developing brain are re-expressed after vestibular injury, constituting the brain orchestration of neurobiological melodies in response to vestibular deafferentation (neurogliosis and neurogenesis, the up-regulation of immediate early genes, neurotrophins, BDNF, and NGF). This early sensitive period for functional reorganization of the neuronal networks implicated in the cat vestibular functions was typical of an adult animal submitted to a total and sudden unilateral loss of vestibular input. The cellular conditions creating such sensitive windows likely vary in magnitude and time with age, the type of vestibular damage (sudden versus progressive, and total versus partial, …), and the type of vestibular function to be recovered (posture, gaze, and navigation, …). Indeed, we showed that the plastic events occurring in the deafferented VN differed considerably depending on the nature of the vestibular input suppression: neurectomy, labyrinthectomy, or reversible blockade of vestibular information by tetrodotoxin (26, 52). Accordingly, these opportunity windows should differ in patients with vestibular neuritis and acoustic neuroma compared with chemical labyrinthectomy and BPPV, with the former group of vestibular pathologies being closer to our animal model. The optimal timing of VR therapy and how VR therapy must be tailored necessitate a better understanding of the cross-talk between retraining procedures and post-lesion brain-plasticity mechanisms (cf. Be Aware of the Instructive Role of Vestibular Rehabilitation).

### Be aware of the instructive role of vestibular rehabilitation

In a paper published 20 years ago, we provided a strong experimental argument in favor of the instructive role of post-lesion experience on the neuronal properties of VN cells (49). In this experiment, the response of well-identified second-order VN cells was recorded in intact cats and in UVN cats housed just after surgery in different visual environments for 1 month, that is, during the whole so-called post-lesion sensitive period. One experimental group was housed in the light, a second one received passive optokinetic stimulation (using the sensorimotor restriction paradigm), and the third group was free to move in the environment and actively received the same optokinetic stimulation as group 2. The results showed that in the control cat, the VN cells responded to visual motion in the low frequency range only (0.1–0.5 Hz), corroborating the general statement that vision intervenes at low frequencies. The groups of cats housed in the light or passively receiving visual information showed no neuronal response changes compared to the controls. However, the UVN cats actively engaged in the optokinetic environment exhibited strongly increased neuronal responses, and at higher frequencies of up to 1 Hz (Figure 1D). These data clearly showed that the VN cells in this latter group were now able to code fast visual motion cues on the basis of visual information, and not on the basis of vestibular inputs as done in the healthy controls. Further experiments with the same paradigm showed that the posturo-kinetic performance of this group of cats was also better compared with the others (58).

This important result can be seen as a visual substitution process at the neuronal level. It also underlies the role of sensorimotor activity in the functional remodeling of the VN network. However, the most important point is that this reorganization depends strongly on the post-lesion experience. Post-lesion interventions and manipulations can therefore modify the neuronal properties of deafferented VN cells, and play an instructive role in functional recovery. In the same line, the newly generated neurons observed in the deafferented VN in the cat model (26, 54) should be validated by post-lesion experiences and integrated into the functional VN network. Further experiments showed that this neurogenesis (and astrogenesis) process played a significant role in the vestibular function recovery (25). Antimitotic drug (AraC) infusions in the VN totally blocked the neurogenesis process and induced a drastic delay of posturo-locomotor recovery (time-course recovery X 3.5), a result obtained only when infusion was performed during the first three post-lesion weeks. Again, the results support the existence of a critical period during which post-lesion experience could tune the functional properties of the newly generated VN neurons.

Translated to the clinic, these data suggest that finding an optimal rehabilitation paradigm after vestibular injury requires an optimal orchestration of the plastic events remodeling the neuronal networks and the VR interventions in accordance with the post-lesion sensitive period. The VR therapy should be initiated during this critical plastic time window characterized by major structural and functional changes. The efficacy of VR therapy might benefit from coinciding with the early stage of plastic events expression, and may contribute to stabilizing, guiding, and/or shaping the newly formed functional connections in the deafferented VN and associated neuronal networks. This is in agreement with the top-down approach to vestibular compensation proposed by Balaban et al. (31). Spontaneous recovery of vestibular functions can be explained by a bottom-up process involving all of the molecular and cellular events in response to vestibular input suppression, and VR therapy can shape and refine the reorganizations in a top-down process through specific interventions and exercises. According to this view, VR therapy could have a leading role, or at least an instructive role in the process of vestibular compensation. However, randomized controlled clinical trials are still lacking to definitively validate this hypothesis. What is true is that the timing, type, and intensity of VR therapy should be crucial factors (see Take into Account the Sensory, Motor and Cognitive Profile of the Patients, Be Careful with Therapeutic Progression, and Reduce Anxiety and Stress). As stated by Merzenich et al. (42), “brain-plasticity based therapeutics can be expected to drive fundamental re-normalizing corrections for distorted brain systems.”

### Favor adaptation rather than habituation processes

How are the vestibular functions restored? Today three different mechanisms of recovery have been reported: restoration, adaptation, and habituation [cf. (19, 59, 60)].

*Restoration* means that the lost function is recovered with the original structural elements and operating mode, before the vestibular damage. That implies for example that the peripheral sensory hair cells can regenerate. This aspect of the vestibular compensation process is currently receiving strong research attention, and data collected *in vitro* indicate that the intrinsic capacities of peripheral vestibular synapses to repair do exist (61). The only indication of such an operating mechanism in humans can be drawn from patients with vestibular neuritis examined with the video HIT. A positive HIT recorded for a given canal at the moment of the disease, which attested to the total loss of function for this canal, was replaced by a negative HIT some times after an acute attack of vestibular neuritis (22). The authors reported the case of one patient with well-identified vestibular neuritis who had a full restoration of his horizontal canal function after weeks and months, as shown by the return of the slow phase eye velocity response to unpredictable head turns to the lesion side and VOR gain close to 1.0, and as it did so, the quick saccades became progressively smaller. Such objective measurements of peripheral vestibular function indicate that the compensation is due to the actual restoration of peripheral vestibular sensory function. Today, this research field needs to define adequate strategies to reconstitute the altered sensory network and restore inner ear function.

Restoration of the VOR in humans with a vestibular neuroprosthesis seems a promising solution in the very near future, and particularly for patients with bilateral vestibular deficits. The vestibular neuroengineering research has recently developed an artificial version of the vestibular system, called the vestibular implant. The external part of the vestibular implant registers head movements with gyroscopes and accelerometers. A microprocessor computes the measurements and transmits results to the internal part, which has electrodes connected to the branches of the vestibular nerve or located within the semicircular canals (62). The technological and physiological feasibility of vestibular implant was investigated and verified in animal models (63–66). The brain appears to adapt plastically to the cues provided via artificial electrical stimulation of the vestibular nerve or ampullary neurons. Research to date includes just a few human studies performed by two teams that work together: the department of ENT at the University Hospital Geneva, and the department of otorhinolaryngology and head neck surgery at the Maastricht University Medical Center. Eye movements were produced in response to electric stimulation of the human posterior ampullary nerve (67) and lateral and superior ampullary nerves (68), and restoration of the VOR was observed with a prototype vestibular neuroprosthesis (69). These important results allow clinicians to envision clinically useful rehabilitation of bilateral vestibular loss patients.

*Adaptation* is usually described in the literature as two separate mechanisms called sensory substitution and behavioral substitution, even though this categorization can differ according to authors. The common characteristic of these two recovery mechanisms is that they constitute learning processes, which are acquired actively and require dynamic interactions of the subject with the environment. In both cases, the lost function is not restored but replaced by a new operating mode using either other sensory cues or a newly elaborated behavioral strategy. This means that adaptation is a qualitative variation of the response, resulting from positive learning.

The vestibular functions are highly dependent on multisensory integration processes that combine vestibular, visual, somatosensory, and haptic cues. They depend on multiple reference frames (geocentric, allocentric, and egocentric). Vestibular loss patients can select a new reference frame for posture control and orientation perception (12). This is done by a re-weighting of the remaining sensory cues. The literature is rich with examples showing that visual cues compensate for the loss of vestibular information and substitute as a reference for Earth vertical in controlling posture and trunk stability (70). Sensory substitution is not, however, a uniform process, but varies widely among individuals (see Do not Use a Stereotyped Vestibular Rehabilitation Protocol). On the other hand, lost dynamic vestibular functions can be replaced by new behavioral strategies involving several neuronal networks distributed in the brain, which reorganize to mimic the lost function (71). The micro-saccades first described in the frog model (72) are a good illustration of a behavioral strategy substituting for normal VOR that aim at maintaining adequate gaze stabilization. More recently, the Australian group described the covert saccades, which are a substitute for the missing dynamic VOR function in vestibular loss patients, and prevent oscillopsies during head rotation at high frequency. The covert saccades maintain near-normal gaze stabilization (11, 18, 73). Again, behavioral substitution had great variability among individuals (see Do not Use a Stereotyped Vestibular Rehabilitation Protocol). Interestingly, the covert saccades were also found when the HIT was performed passively (21, 22). What triggers the covert saccades in passive conditions remains an open question. However, these authors suggest that the covert saccades could be produced by neck afferents being triggered at the very start of the head turn and not as the result of an increased gain of the cervico-ocular reflex (COR), which would generate slow phase eye velocity. Potentiation of the COR may be valuable for passive low acceleration head movement, but not during abrupt high acceleration head turns.

*Habituation* differs from adaptation for several reasons. Strictly speaking, habituation is the progressive reduction of a response due to the monotonous repetition of the same stimulus, until the response totally vanishes. It is acquired passively (it does not need active training), and it represents a quantitative variation of the response (not a qualitative variation). The goal for habituation processes is “do not respond” instead of “respond differently” for the adaptation processes. From a physiological point of view, the mechanisms underlying habituation are totally different from those responsible for adaptation. The acquisition of habituation is due to a decrease in the excitatory post-synaptic potentials as a result of calcium channel blockades at the pre-synaptic level, while retention of habituation is due to the expression of second messengers and the synthesis of new proteins modifying the structure of the synapses (Hebbian plasticity). VR therapy based on habituation exercises is not so commonly used. The Habituation Training Program elaborated by Norré and Becker (74) for vestibular loss patients does not really fit with the habituation concept because the exercises they proposed are performed actively by the patients, thus activating both feedback and feed forward mechanisms. The same is true for the Brandt and Daroff exercises elaborated for the treatment of the BPPV (75). These VR therapies more likely constitute desensitization practices, that is, the learning to tolerate abnormal or disturbing responses. They have a place in the treatment of many symptoms including visual dependence and visual vertigo. Including optokinetic stimulation in a VR protocol can be a good way to reduce visual dependence (76, 77).

Vestibular rehabilitation therapy should focus on adaptation, particularly for the restoration of the dynamic functions. Sensory substitution is a powerful tool for compensating vestibular loss, and it is relatively easy to increase the remaining inputs by manipulating the visual cues (eyes open, eyes closed, and optokinetic stimulation), by manipulating balance control (on stable surfaces, on foam, and on unstable surfaces), and by combining both protocols. Sensory addition is another tool. Sensory biofeedback based on various sensory inputs can be helpful in a VR protocol (70, 78). Augmenting sensory information by providing visual, auditory, and somatosensory input has been shown to reduce postural sway during stance and gait (79, 80). The electrotactile vestibular substitution system (BrainPort) is a new treatment modality in vestibular loss patients. It is a human–machine interface that transmits information about patient’s head position via electrotactile stimulation of the tongue (81). The efficacy of the BrainPort balance device has been observed in bilateral vestibular loss patients (82) and in patients with peripheral and central vestibular loss (83). Significant improvements in patient’s symptoms (the DHI questionnaire), posture stability (the computerized dynamic posturography system), and gait (the dynamic gait index test) were reported. Balance improvement in patients with vestibular disorders has also been obtained through instrumental rehabilitation with new moving platforms providing predictable perturbations (84–86). Such powered platforms allow the patient to rely not only on feedback mechanisms for control of posture, but also on feed forward mechanisms: the patient learns to anticipate the appropriate muscle responses to counteract the balance perturbations.

### Do not use a stereotyped vestibular rehabilitation protocol

The pathophysiology of vestibular disorders is so varied that it is not conceivable to have a unique VR protocol for all patients. In addition, the sensory and behavioral strategies used for recovery are so different among individuals that a unique protocol would be non-sense. The recovery of balance control in static and dynamic conditions as well as the recovery of gaze stabilization is achieved differently among patients, indicating that vestibular compensation constitutes a vicariant idiosyncratic process, with each individual finding his/her own solution to vestibular loss-induced problems.

In their studies of ocular stability during active eye–head turning in normal monkeys, Bizzi et al. (87, 88) investigated the relative contribution of vestibular and neck afferents to the compensatory eye movements. They showed that at least 95% of ocular stability was due to the vestibular loop, and that the contribution of the neck loop was negligible. They were also the first to investigate the mechanisms underlying recovery of eye–head coordination following bilateral labyrinthectomy in monkeys (89). These authors described that there were at least three mechanisms underlying the recovery of compensatory eye movements, which reaches 90% within 7 weeks: an increase in gain of the neck loop, the occurrence of centrally programed compensatory eye movements, and a recalibration of the saccadic and head motor system. The major contribution of the saccadic system to compensatory eye movements was demonstrated thereafter in other animal models [(72): frog] and in vestibular defective human beings (90). The covert saccade discovered by the Australian group and evoked previously is a behavioral strategy able to restore near-normal dynamic visual acuity in unilaterally vestibulopathic humans (91) as well as patients with bilateral vestibular hypofunction (92). However, the behavioral strategies substituting for the lack of dynamic VOR function vary considerably among patients. Some patients close their eyes during head rotation and open their eyes at the end of head rotation, thus avoiding any retinal slip and oscillopsia. A variation of this strategy is to perform several blinks during head rotation (Michel Toupet, personal communication), a strategy that samples eye positions, with the brain reconstructing head motion. Another strategy is to slowly move the whole body as a block. In this case, the optokinetic reflex is activated and gaze stabilization is achieved, at least in the low frequency range, which unfortunately does not correspond to the normal range of head displacement. Finally, using fMRI investigations in UVN patients, a German group described a strong deactivation of the visual cortex during optokinetic stimulation (93). Suppression of cortical visual motion processing during head rotation could constitute another strategy used for suppression of the perception of retinal slips. Such idiosyncratic strategies, depending on the patients themselves, have been observed in the cat model (53). Impaired eye–head synergy was recovered in 50% of the UVN cats by changing the temporal pattern of neck muscle activity without re-weighting of the visual cues (behavioral substitution by the coactivation of neck muscles) while the second half of the population increased the visual input weight without modifying the neck muscle activation pattern (pure visual substitution process).

Patients’ recovery of gaze and balance is achieved by vicarious idiosyncratic strategies, a concept that has direct implications for VR therapy. The VR physiotherapist first has to suppress the maladaptive strategies observed in their patients. Avoidance strategies that limit head movements, as well as the use of blinks during head rotation that limit visual perception, must be suppressed. Opening the eyes and keeping the head free in space during head rotation to the injured side are instructions to provide to the patients. VR therapy must also unlearn these maladaptive strategies and help to find more adaptive strategies using exercises based on learning. The dynamic visual acuity test should be used to more or less perfectly restore the impaired VOR and the patient’s ability to perceive objects accurately while actively moving his/her head (94–97). The VR physiotherapist also has to check the sensory strategy used by the patients and to adapt their protocols accordingly (see Take into Account the Sensory, Motor and Cognitive Profile of the Patients).

### Take into account the sensory, motor, and cognitive profile of the patients

As previously reported, most of the vestibular functions are achieved on the basis of multisensory integration processes described at all levels of the vestibular pathways, from the VN to the thalamus and cortical areas (the parieto-insular cortex, and areas 3a, and 2v) considered to be the multi-site vestibular cortex [see Lopez and Blanke (98) for review]. Visual and somatosensory cues as well as the remaining vestibular inputs from the intact side are good candidates to substitute for the missing vestibular information on the diseased side.

Investigations on posture control using static and dynamic platforms have pointed to the substitution role of these different sensory cues. Creath et al. (99) reported that the best compensated patients with unilateral vestibular loss, regarding postural orientation and stabilization, were those who used their remaining vestibular function, sometimes as much as controls with intact bilateral vestibular function, while others did not. In Menière’s patients, who were submitted to a UVN, two different post-lesion sensory strategies for balance control were observed (100). Half of the subjects used their vision and had a better postural performance with their eyes open compared to with their eyes closed (visual strategy), while the other half had less instability with their eyes closed (somatosensory or proprioceptive strategy). However, too strong a reliance on vision can create a visual dependence that will impair the recovery of balance control (77). Another important point is that sensory strategies are typical of particular contexts, and can change in other environmental conditions. Menière’s patients were found to shift from one spatial reference frame to another, from internal to external references or vice versa, when manipulating the visual world (101). In severe bilateral vestibular patients after ototoxic loss, the postural performance of some subjects was better than others when using their vision (102), haptic cues (99), or proprioceptive inputs (103). However, too large a reliance on substitution information can impair balance performance. Poorly compensated vestibular patients exhibited great instability because their proprioceptively triggered responses were too large (103), a result already reported in bilateral labyrinthectomised cats (104).

Taken together, these data show that sensory cues substituting for the missing vestibular information vary widely among individuals. Because different reference frames are used for body orientation and stabilization, VR therapy must be customized for each patient. The behavioral and cognitive aspects should also be checked. Physiotherapists should determine the sensory profile, or perceptive style, of their patients to adapt their VR therapy by incorporating the most adequate exercises in their protocol. The patient’s sensory profile can be determined with different tools such as the SVV test, static and dynamic posturography, and manipulation of the sensory contexts (balance control on stable/unstable platforms, with or without foam, and with eyes open, closed, or during optokinetic stimulation). Rehabilitation programs of balance recovery should include the SVV perception test. This simple test may disclose tilts or verticality misperceptions due to otolith or vestibular pathway impairment (105) as well as stroke (106). The behavioral strategy the patients use can be determined by direct examination and their cognitive style can be assessed by different questionnaires (the DHI test, and anxiety and depression scales). Physiotherapists also have to fight a too strong dependence on particular input by desensitization maneuvers.

### Be careful with therapeutic progression

Today, there are no generally accepted guidelines and no definite recommendations concerning the different facets of VR therapy, that is, timing, type, intensity, and duration. In addition, VR therapy protocols should depend on the nature of the vestibular deficit and on the sensorimotor and cognitive profile of the patients, which are other reasons why it is difficult to propose a unique protocol.

Nevertheless, general principles and recommendations must be proposed. Most of them have been summarized in a recent review (31). According to these authors, therapeutic progression must proceed from the head to locomotion in a top-down strategy of exercise progression (107, 108). Eye and head movements will first be performed in the seated subject, if the subject is not able to stand safely, and in static postural conditions if an upright posture is possible. Stability limitations will be observed in more challenging situations, with the eyes closed, on foam, and with increasing rotation speeds of the head. Dynamic gait rehabilitation will follow using the same progression pattern. VR therapy must be seen as a sequential process with intermediate goals, or as a stepwise learning process (31).

The VR therapy must impact all facets of the vestibular syndrome, that is, the postural, oculomotor, and perceptive symptoms following vestibular injury, in addition to the associated neurovegetative symptoms that must first be addressed. Prior to VR therapy, nausea, vomiting, and vertigo must be stopped or reduced. Antiemetics and anti-vertigo drugs can be useful in the early acute stage, that is, 2–3 days after vestibular injury, and should then be stopped. Spontaneous nystagmus will be reduced by eye fixation exercises. The rehabilitation exercises must target all sub-systems sub-serving dynamic gaze stabilization, balance control, and spatial perception. Regaining eye motion control and eye–head coordination are primary goals of VR therapy (107, 108). The dynamic VOR recovery is limited in part due to the restriction of head movements (the head on trunk stiffness strategy that has to be unlearned), and exercises based on active head motion in space will be performed for gaze recovery in a static posture, and then in more dynamic challenging conditions, such as while walking, hopping, and running.

The incorrect progression of VR exercises may lead to poor recovery and/or long-lasting rehabilitation therapy interventions. The physiotherapist must question the validity of the VR therapy when a patient still has important complaints after 100 training sessions, as we have sometimes observed. It is only in private dialog with the patient that the physiotherapist will determine the underlying reasons for the failure of the VR therapy.

### Reduce anxiety and stress

The vestibular syndrome resulting from vestibular injury alters homeostasis and initiates an adaptive stress response, which in turn induces the release of glucocorticoids via the activation of the hypothalamo-pituitary-adrenal (HPA) axis. Stress-related hormones have been found in different animal models. HPA activation was observed in vestibular deafferented pigmented guinea pigs (109). Our findings in the UVN cat showed an up-regulation of Fos expression underlying increased cell activity in the paraventricular nucleus (PVN) of the hypothalamus (53), a neuroendocrine plasticity mechanism that supports the adaptive response to vestibular loss-induced stress. This was confirmed by modifications of the regulation of corticotrophin-releasing factor and arginine vasopressine in the PVN and the VN (56). The release of glucocorticoids is known to contribute to the neurochemical mechanisms of vestibular compensation (110), and agonists and antagonists of the glucocorticoid receptors accelerate and slow postural compensation, respectively (111).

Interestingly, a long-lasting activation of the HPA axis was found in our cat model (56). This could reflect chronic stress during the whole compensatory period, lasting until the animal remained totally free of vestibular symptoms. Whether stress is detrimental or not remains an open question in the literature, but it is very likely that a high level of stress would impair functional recovery. Vestibular injury constitutes a stressor signal, and stressed patients behave differently than unstressed subjects, particularly in challenging situations in which they do not engage behaviorally in the same way. Reducing stress in vestibular loss patients by using behavioral and/or cognitive therapies should be a goal of physiotherapists.

Anxiety and depression are other psychological factor that can interfere in the compensation process; they must be taken into account in a VR protocol. Using the hospital anxiety and depression scale, it was found that patients with vertigo had significantly higher scores compared with healthy controls (112). We used evaluation questionnaires to examine the compensation level of vestibular loss patients (the Dizziness Handicap Inventory test), their general anxiety level (the Short Anxiety Screening Test), and their fear of height and avoidance of risky situations (a subjective scale). The patients showed higher scores compared with control subjects when examined a long time after vestibular injury (30). The positive effects of VR therapy based on exercises aimed at improving the VOR gain and balance control have been described by the emotional aspects of chronic vestibular deficit patients (113). Again, physiotherapists must keep in mind the psychological profile of their patients, and in case of severe anxiety disorders or panic disorders, the anxiety and depression should be treated first. Moreover, anxiety and stress can impair cognitive functions by inducing structural modifications such as dendritic atrophy and spine reduction, notably in the hippocampus (114) and nervous structures linked to the VN [locus coeruleus and the dorsal raphe nucleus, see in Ref. (115, 116)]. Spatial navigation, for example, is one of the cognitive aspects of vestibular compensation that can be sensitive to acute and chronic stress. Deficits in path integration were observed in unilateral vestibular loss patients, which could not result from the vestibular deficit alone (117).

De-compensation, a phenomenon referring to patients again experiencing, partially or totally, the vestibular syndrome a long time after compensation (vertigo, instability, and nystagmus), can be induced by highly stressful situations. This has been observed in labyrinthectomised rats placed in weightlessness situations (118). Some compensated vestibular loss patients reported the de-compensation phenomenon in supermarkets or in crowded streets and in situations in which moving visual scenes are present. The physiotherapist must inform patients of this potential de-compensation phenomenon, particularly after alcohol consumption and the use of sedative drugs.

### Favor ecologic situations and contexts

Because the ultimate goal of VR therapy is to regain a good quality of life, VR therapy protocols must incorporate interventions and exercises that patients encounter in most of their daily life situations. Another reason to promote the use of more natural contexts is the difference often noted between the standardized outcomes under unattractive laboratory situations and the functional gains for daily life activities. Moreover, enriched environments have been shown to represent significant therapeutic potential by inducing neuroprotective mechanisms that improve behavioral outcomes after experimental brain injury [(119); cf. Motivate the Patients). The role of enrichment in remediating brain injury in animal models is very well known. Environmental enrichment results in a number of molecular and morphological alterations, which are thought to underpin changes in neuronal functioning and behavior (44, 45).

In most cases, clinical settings after traumatic brain injury include multi-modal stimulation, physical and cognitive therapy that can be seen as enriched environments. VR protocols should use such enriched ecologic contexts as paradigms that enhance and promote engagement with cognitive, social, and physical stimulation. Functional relevance, duration, timing, novelty, and complexity are other components of environmental enrichment that contribute to better general functional outcomes. Much work has still to be done, however, to clarify our understanding of environmental enrichment as a therapeutic tool after brain injury in humans [see Alwis and Rajan (45) for review].

Virtual reality is a relatively new technology that can reproduce daily life situations and contexts that are more motivating for patients. It creates an immersive experience or a sense of three-dimensionality such that subjects believe that they are moving within a realistic world. This technology is used to enhance perception, elicit automatic motor responses, and increase sensorimotor adaptation (120). Similar to prism adaptation, virtual reality technology can be used to alter the egocentric and allocentric representations of the world. It has been included in NASA programs to decrease motion sickness susceptibility in astronauts during microgravity space flights, and to facilitate their behavioral recovery when returning to 1 g gravity (121). The beneficial effects of virtual reality were also reported in patients with peripheral vestibular dysfunction exhibiting visual vertigo symptoms (122). However, this sophisticated technology is costly and thus cannot be used by the whole community of practitioners. Other limitations include virtual reality-induced anxiety and fear, particularly in older subjects. For aged patients, Tai Chi exercises can be recommended, notably for improving balance control and reducing the risk of falling (123, 124). Playing tennis or badminton and dancing are more natural exercises requiring a high level of dynamic eye–head coordination at a lower cost. General activity exercises performed daily at home (walking and cycling) are also recommended.

The dual-tasking condition is very common in most day life conditions, and it represents another ecologic context in which a simple postural task is paired with cognitive activity (talking while walking). Competition for attentional resources is a consequence of simultaneously performing balance tasks and cognitive tasks, and age-related changes in posture control were reported in healthy subjects (125–127). Interference between postural control and mental task performance was also observed in patients with vestibular disorders (29). The postural performance decrement observed in vestibular loss patients during dual-tasking likely reflects their inability to share attentional resources between the two tasks. By focusing their attention on the postural task [the “posture first principle” described in older adults under dual-tasking situations; see Shumway-Cook et al. (125)], vestibular patients use maladaptive behaviors like the “stiffness-like” strategy (30) and exhibit poor postural performance. VR protocols should systematically incorporate the dual-tasking paradigm because of the cross-domain attentional resource competition that it creates.

### Motivate the patients

A patient who walks backwards for VR therapy sessions has limited chances to recover fast and optimally. Patient motivation is a crucial factor that the physiotherapist has to take into account and to promote.

Many recently developed devices allow intense training without continuous and costly physiotherapy assistance. Modern set-ups and training devices are combined with biofeedback games or video games that can boost the motivation of the patient. An example is the use of static or dynamic platforms on which the patient is asked to maintain equilibrium, while looking at a screen on which are displayed his/her own center of gravity together with colored balloons appearing at different locations. The “game” consists of reaching the balloons with the image of the center of gravity, by moving the whole-body leftward/forward or leftward/rightward. A score can be given for each session, and the patient can see the improvement of his/her performance session after session. Training presented in game-like training formats on mobile devices or computers is another way to boost motivation for VR therapy. The Nintendo Wifi balance Board has been used in VR protocols (128). While described as a friendly alternative, our feeling is that it does not constitute a real new perspective for the future.

The use of a mental imagery training procedure could be another paradigm to improve vestibular compensation and to motivate patients who restrain their head and body movements after a vestibular deficit. Motor imagery has been applied in rehabilitation programs of neurological patients after stroke and some studies reported a beneficial influence on motor functions (129, 130). Neuroimaging studies reported that the efficacy of motor imagery in post-stroke rehabilitation was based on overlapping neural networks involved in both imagined and executed body movements (131). It can be postulated that integrating imagined whole-body movements in a VR protocol can improve functional recovery (132). This hypothesis is based on functional MRI investigations showing that imagined self-body rotations activate the temporo-parietal junction and the posterior parietal cortex (133), that is, cortical areas overlapping with the human vestibular cortex (134).

Ecologic situations are also more motivating for the patient than unattractive laboratory tools. And as discussed previously (see Favor Ecologic Situations and Contexts), environmental enrichment-based rehabilitation is another solution to motivate patients, to enhance functional recovery, and to obtain a good quality of life.

## Conclusion

This review aimed to provide physiotherapists with an understanding of the brain-plasticity mechanisms responsible for spontaneous recovery after vestibular injury, and of the interaction that VR therapy may have with basic recovery mechanisms. It highlights the critical plastic time window of internal processes of reorganization and of VR interventions. VR therapy is indicated in any form of vestibular injury, including BPPV (a particular form of VR therapy), chronic poorly compensated patients, and aged and dizziness subjects, as well as patients with visual vertigo and motion sickness. There is not one optimal VR therapy *per se* because functional recovery depends on so many intrinsic (vestibular pathology, age, motivation, and anxiety) and extrinsic (environmental context) factors. This review provides clinicians with common sense principles and recommendations for achieving their patient’s best recovery in every daily life situations and for regaining a good quality of life. VR therapy paradigms that enhance engagement with physical, cognitive, and social stimulation and that include novel and multi-modal stimulation result in better general functional outcomes. Active and early VR therapy is crucial, as well as the progression of the exercises in the protocol, which has to be patient-dependent by taking into account the sensorimotor, cognitive, and emotional profiles of the individual patients. Poor compensation can be explained in part by maladapted VR therapy protocols, and the de-compensation process might be explained by anxious and stressing environments. The outcomes of VR therapy should be corroborated by both subjective self-evaluation by the patient and objective laboratory measurements.

## Conflict of Interest Statement

The authors declare that the research was conducted in the absence of any commercial or financial relationships that could be construed as a potential conflict of interest.
